# Persistence of *Salmonella* and *Campylobacter* on Whole Chicken Carcasses under the Different Chlorine Concentrations Used in the Chill Tank of Processing Plants in Sri Lanka

**DOI:** 10.3390/pathogens13080664

**Published:** 2024-08-07

**Authors:** Gayani Weerasooriya, H. M. T. Dulakshi, P. S. de Alwis, Sandun Bandara, K. R. P. S. Premarathne, Nayanajith Dissanayake, N. Liyanagunawardena, M. I. Wijemuni, M. A. R. Priyantha

**Affiliations:** Bacteriology Division, Veterinary Research Institute, Peradeniya P.O. Box 28, Sri Lankashyamalidealwis@gmail.com (P.S.d.A.); pasindushaneth9@gmail.com (K.R.P.S.P.); wijemunimadhavi@gmail.com (M.I.W.); madalagamaroshan@gmail.com (M.A.R.P.)

**Keywords:** *Campylobacter*, *Salmonella*, chlorine, chicken meat, MDR

## Abstract

The persistence of non-typhoidal *Salmonella* and *Campylobacter* in chicken meat is a considerable public health risk and a future challenge. This study aimed to determine the prevalence of *Salmonella* and *Campylobacter* in poultry processing lines where different chlorine concentrations were used in the chill tank. The samples were collected from four types of processing plants in Sri Lanka, considering the chlorine concentration used in the chill tank, which ranged from 2 ppm to 50 ppm. *Salmonella* and *Campylobacter* were isolated from whole carcass washings, neck skin, and cecal samples. Subsequently, an antimicrobial susceptibility test was performed for the isolates. The results revealed the overall prevalence of *Salmonella* and *Campylobacter* was 78.25% and 63.5%, respectively. Positive percentages of *Salmonella* and *Campylobacter* were high in the carcasses compared to the neck skin and ceca. The *Campylobacter* counts on the whole carcasses were significantly low (*p* < 0.001), at higher chlorine concentrations ranging from 20 to 30 ppm and 40 to 50 ppm. The pathogen prevalence in the whole carcasses was 84.7% *Campylobacter coli*, 39.1% *Campylobacter jejuni*, 71.1% *Salmonella* Typhimurium, and 28.8% *Salmonella* Infantis. The highest resistance was observed for tetracycline (63.8%) in *Salmonella*, while it was for gentamicin (87.8%) in *Campylobacter*. The prevalence percentage of multidrug-resistant *Campylobacter* was 51.2%, while it was 2.12% for *Salmonella*. The persistence of multidrug-resistant *Salmonella* and *Campylobacter* on the post-chill carcasses was highlighted in the present study as a significant public health threat that has to be addressed urgently.

## 1. Introduction

Foodborne infections are a major public health issue with a significant socioeconomic impact on the globe [1]. The World Health Organization (WHO) has reported that contaminated food resulted in 600 million cases of illness and 420,000 deaths worldwide [2]. *Campylobacter* and *Salmonella* species are identified as leading causes of acute bacterial gastroenteritis in humans [3]. According to the Food Net Surveillance Data System in the United States, out of reported bacterial gastroenteritis cases, 34.6% were caused by *Campylobacter* while 37.9% were caused by *Salmonella* spp. [4]. As both pathogens are colonized in the chicken gut, consumption of contaminated raw or undercooked chicken meat and their by-products may cause gastrointestinal infections in humans [5,6]. Although possible interventions were implemented in the poultry industry to limit the bacterial contamination in the ‘hygienic farm to fork’ concept, the contamination of chicken meat with foodborne pathogens was still significant. To strengthen Good Management Practices (GMPs) in the production chain, the USDA Food Safety and Inspection Service (FSIS) has introduced performance standards: Hazard Analysis and Critical Control Point (HACCP), for *Campylobacter* and *Salmonella,* safeguarding public health by ensuring food safety throughout the food chain [7]. Physical and chemical interventions such as using either hot or cold water in carcass washers, with or without sanitizers, are applied to mitigate foodborne pathogen contamination [8]. According to the surveillance data, the *Campylobacter* contamination percentages of post-production poultry meat varied between 10% and 100% depending on geographical location [9]. In the USA, a surveillance study has shown that the prevalence of *Salmonella* was 4.2% and *Campylobacter* was 70.5% in raw chicken meat [10]. In Australia, the presence of *Campylobacter* and *Salmonella* in raw chicken meat was estimated as 84.3% and 22.1%, respectively [3]. In Asia, the recovery percentages for *Salmonella* in chicken meat varied from 35% to 50%, and limited information was found for *Campylobacter* [11,12].

Chicken meat is the most consumed meat commodity in Sri Lanka, due to its low cost and easy availability [13]. The meat industry still plays an important role in the livestock sub-sector of Sri Lanka and chicken meat contributes about 70% and is the only meat exported [13]. The per capita availability of chicken meat was 10.3 kg in 2021 [14]. The local whole chicken production in 2022 was reported as 228,130 metric tons. The production of chicken meat-based products, such as chicken sausages, chicken meatballs, and further processed chicken, in 2022 was 19,545.64 metric tons [15]. Notably, Sri Lanka started poultry meat exportation in 2020, and 502.76 metric tons were exported to Middle East and South Asian countries in 2022 [13]. Poultry meat is produced mostly in large-scale semi-automated processing plants with 20,000–30,000 birds/day processing capacity, operating under GMPs and HACCP standard methods. As in other countries, using chlorinated water in the inside–outside carcass washers and the chill tank are the most common chemical decontamination practices that have been implemented to mitigate bacterial contamination during processing in Sri Lanka. However, according to the few studies conducted, the prevalence of foodborne pathogens in chicken meat is considerably high [16]. The entry of a high bacterial load into the processing line, due to the high prevalence of pathogens in poultry farms, along with inadequate chemical decontamination techniques in chill tanks during processing, could be some possible reasons for contamination [17]. Nevertheless, the fewer reported foodborne human outbreaks in Sri Lanka, compared to developed countries, could be due to the lower consumption of the bacterial load caused by differences in eating habits and traditional cooking practices such as a longer cooking time under high heat with high concentrations of spices. Furthermore, the misdiagnosis of infections due to less laboratory capacities, limited human resources, and under-reporting of infections due to less awareness might be the other reasons for fewer documented outbreaks in Sri Lanka. Notably, drastic changes in lifestyle, cooking patterns, and eating habits in Sri Lankans during the last few decades might increase the consumption of partially cooked chicken meat. Therefore, it is essential to understand the level of foodborne pathogen contamination in chicken meat in Sri Lanka under the current processing techniques. Although prevalence studies have been conducted in the retail market and fewer selected processing plants [16,18], there is no evidence of comprehensive studies being conducted to include all the major large-scale processing plants in Sri Lanka. With the presence of foodborne pathogens in raw chicken meat, the risk of persisting antimicrobial-resistant foodborne pathogens plays a crucial role in the One Health aspect. *Salmonella* and *Campylobacter* infections can result in a systemic disease requiring the use of antimicrobials [19]. These two foodborne pathogens can be colonized at the same time in the chicken gut, and exposed to the same kind of antimicrobials, which are commonly used in the poultry industry [20,21]. Erythromycin is considered the first-line treatment, and fluoroquinolones are also frequently used due to their broad-spectrum activity against enteric pathogens [22]. Recently, multidrug-resistant *Campylobacter* strains have been detected in poultry and several other sources of meat in the world [23,24,25]. Although the antimicrobial resistance of *Campylobacter* has been reported previously in the limited studies [16], a comprehensive study has not been conducted yet in Sri Lanka. The antimicrobial resistance and multidrug resistance (MDR) in *Salmonella* in broiler meat are well revealed [26]; nevertheless, the resistance to common antimicrobials in *Salmonella* and *Campylobacter* isolated from the same whole chicken carcasses was not studied together. Further, as *Salmonella* and *Campylobacter* can be colonized in the intestine together, it is important to understand the antimicrobial-resistant pattern in these two pathogens.

Notably, being an attractive tourist destination, and a growing chicken meat exporter, it is very important to know the prevalence and behavior patterns of foodborne pathogens in the food chain. Therefore, this in-plant study was conducted with the first objective of determining the prevalence of non-typhoidal *Salmonella* and *Campylobacter* in the processing line in large-scale processing plants under different chlorine concentrations in the chill tank. Moreover, the second objective of the present study was to determine the antimicrobial susceptibility of *Campylobacter* and *Salmonella* isolated from whole chicken carcass washings.

## 2. Materials and Methods

### 2.1. Experimental Design

A survey was conducted before the present study, covering all the large-scale poultry processing plants in Sri Lanka. The raw data were collected on the processing capacity, method of evisceration, availability of inside–outside carcass washers, chlorine concentration used in the chill tank, the temperature of the chill tank water, and the chilling time of a carcass. From the information obtained, ten (*n* = 10) large-scale processing plants were selected for the study, which have similar processing capacities ranging from 15,000 to 25,000 birds per day (60% of total broiler production in the country represented by these selected processing plants). All the processing plants selected were operated in a semi-automated system, where manual evisceration is practiced. After evisceration, carcasses were washed by inside–outside carcass washers. The chill tank temperature of all the selected processing plants was 4 °C and the chilling time was 45 min. With that, we were able to minimize the effect of the chilling temperature and the contact time in this study of the selected processing plants. Only the chlorine concentration used in the chill tank was different and accordingly, three different types of processing plants were identified (A, B, C). Type A: chlorine at 3–5 ppm, Type B: chlorine at 20–30 ppm, and Type C: chlorine at 40–50 ppm. Additionally, Type D: Other (sodium metabisulphite at 200 ppm). All the processing plants except in Type D used chlorinated potable water (2–3 ppm) in carcass washing (inside–outside carcass washers). Cecal samples (*n* = 100), neck skin samples (*n* = 100), and whole carcass washings (*n* = 150) were collected from three different locations of the processing line to isolate *Salmonella* and *Campylobacter*. The samples were collected from the first batch that was processed on the day to minimize the effect of contamination due to other batches being processed. Cecal samples were collected at the evisceration section before the carcasses reached the inside–outside carcass washer, the neck skin samples were collected after the inside–outside carcass washer, and the whole carcass washing was collected at the post-chill stage. The whole carcass washing was collected from the same batch from which the cecal samples and the neck skin samples were collected. Chlorine concentrations, the weight of the carcasses, and the chill tank temperature were recorded at the time of sampling.

### 2.2. Enumeration of Salmonella and Campylobacter

Cecal samples were collected directly from ten (*n* = 10) individual carcasses after evisceration and placed in the sterile bags separately. Ten (*n* = 10) neck skin samples were collected from the same carcasses from the evisceration line after entering through the inside–outside carcass washer and placed individually in sterile 5 in × 6 in polythene bags. Subsequently, fifteen (*n* = 15) individual carcasses from the same batch were collected from the chill tank and placed in 15 in × 12 in sterile polythene bags and washed with massaging for 2 min in 200 mL of sterile distilled water. The carcass wash was collected into 50 mL sterile microcentrifuge tubes. The average weight was recorded for all fifteen carcasses. All the samples were stored in ice until transport to the laboratory.

*Salmonella* and *Campylobacter* were isolated according to the method described by Chousalkar et al., 2019 and ISO 6579-1:2017 guidelines [27,28]. Two hundred micro liter (200 µL) of carcass wash was inoculated into modified charcoal cefoperazone deoxycholate agar (mCCDA; Thermo Scientific, Oxoid, UK) plates by spread plating and incubated at 42 °C with 10% CO_2_ for 48 h to assess direct *Campylobacter* spp. counts. From the initial 50 mL of carcass wash, 10 mL was added to 90 mL of 1% buffered peptone water (BPW; Thermo Scientific, Oxoid) and incubated for 18–20 h at 37 °C. Ten grams (10 g) of neck skin samples were homogenized separately in 90 mL of 1% buffered peptone water, 200 µL of serial 10-fold dilutions of carcass wash was inoculated into mCCDA by spread plating and was incubated at 42 °C with 10% CO_2_ for 48 h to access direct *Campylobacter* counts. The rest of the carcass wash was incubated for 18–20 h at 37 °C for *Salmonella* isolation. Ten grams (10 g) of the cecal content was taken into the sterile polythene bag and 90 mL of 1% buffered peptone water was added and homogenized. Subsequently, 100 µL of serial 10-fold dilutions of the sample was inoculated into mCCDA by spread plating and incubated at 42 °C with 10% CO_2_ for 48 h, to detect the *Campylobacter* count. Subsequently, the rest of the carcass wash was incubated for 18–20 h at 37 °C for *Salmonella* isolation. From the incubated carcass wash, neck skin, and ceca, 100 µL was transferred into 10 mL Rappaport Vassiliadis soya peptone broth (RVS; ThermoScientific, Oxoid) and incubated for 18–20 h at 42 °C for selective growth of *Salmonella enterica* serovars. A loop-full of the RVS broth was streaked onto xylose lysine deoxycholate agar (XLD; Thermo Scientific, Oxoid) plates. Suspected *Salmonella* colonies were sub-cultured onto Colombia sheep blood agar (Thermo Scientific Oxoid) and the biochemical tests, TSI, Urease, Citrate, and SIM, were performed for confirmation. For *Campylobacter* confirmation, Gram stain smears were prepared to identify the specific spiral morphology of the bacteria from the suspected colonies in mCCDA. Subsequently, an oxidase test was performed to observe positivity. For *Campylobacter* spp., the limit of detection was 10 CFU/mL of carcass wash.

### 2.3. Molecular Identification of Campylobacter and Serotyping of Non-Typhoidal Salmonella

The species identification for *Campylobacter* was performed for the whole carcass *Campylobacter* isolates using real-time polymerase chain reaction (qPCR). DNA was extracted from the 500 µL of broth cultures by the boiling technique described previously [29] and quantified by a NanoDrop spectrophotometer (ThermoScientific). The qPCR test protocol was performed based on the procedure used elsewhere [30,31], using SYBR Green Master Mix with some modifications (the volume of Master Mix and nuclease-free water were changed according to the reaction volume used). The primers used in this analysis were aimed at identifying the *hipO* gene for *C. jejuni* and the *glyA* gene for *C. coli* (Table 1).

The PCR mixture consisted 25 μL of 15 μL SYBR Green Master Mix (Quagen, USA), 1 μL (10 pmol) of the forward and reverse primers of each gene (DIT, USA), 6 μL of nuclease-free water, and 2 μL of the DNA template. DNA from *C jejuni* ATCC 33,291 and *C. coli* ATCC 33,311 were used as the positive controls and nuclease-free water without any DNA was used as the negative control. The qPCR amplification was performed on a thermal cycler Bio Rad CFX Maestro (Bio Rad Laboratories, Hercules, CA, USA). The amplification program consisted of the initial denaturation at 95 °C for 3 min, followed by 40 cycles of denaturation at 95 °C for 3 s, annealing at 60 °C for 30 s, and extension at 72 °C for 20 s [30,31]. The primer efficiency was determined by preparing standard curves. Real-time data were analyzed with CFX Maestro Software (version 2.3). Compared to the quantification cycle (Cq) value for the specific genes in the positive control, the Cq values were obtained from the sample to determine the species of *Campylobacter.* Cq values more than or similar to the negative control were considered as negative. All the samples were run in duplicates.

*Salmonella* species identification was performed by serotyping using a panel of polyvalent antisera (Microgen Bioproducts, UK) according to the Kauffman–White scheme [32] for whole carcass *Salmonella* isolates. *Salmonella* isolates were sub-cultured once to obtain pure cultures, stored at −80 °C in 50% glycerol. The slide agglutination test was performed for the common poultry non-typhoid *Salmonella*. The smears were prepared on the microscopic glass slide using sterile normal saline. A drop of antisera was added to the smear, mixed well, and the agglutination was observed while rotating the slide (Table 2).

### 2.4. Minimum Inhibitory Concentration (MIC) for Campylobacter and Salmonella

A micro broth dilution test was performed to determine the MIC for four clinically relevant antibiotics (Sigma-Aldrich): gentamicin, ciprofloxacin, nalidixic acid, and tetracycline as described by the Clinical and Laboratory Standards Institute (CLSI) and European Committee on Antimicrobial Susceptibility Testing (EUCAST) [33,34,35]. Briefly, the inoculum was prepared from the overnight grown forty-one (*n* = 41) *Campylobacter* isolated from the whole carcass washing on sheep blood agar, which was stored at −80 °C in 50% glycerol. The inoculum concentrations were prepared to 0.5 McFarland Standard by suspension in sterile distilled water. Ninety microliters (90 µL) of Nutrient Broth No 2 (Thermo Scientific, Oxoid) was added into columns 2–12 of 96-well microtiter plates. The stock solutions of antimicrobial solutions were prepared in sterile distilled water and 180 µL was added into the first column and the two-fold dilutions were performed to obtain gentamicin (0.031–32 µg/mL), ciprofloxacin (0.076–16 µg/mL), nalidixic acid (0.125–256 µg/mL), and tetracycline (0.076–16 µg/mL). Separate microtiter plates were used for separate antimicrobials. Subsequently, 10 µL of the prepared inoculum was added into all the wells of each row and incubated at 42 °C with 10% CO_2_ for 20 h. Likewise, eight inoculums were performed in one plate with one antimicrobial type. Sterility control and antimicrobial control were used in every test performed.

The same method was used to determine the MIC for the whole carcass washing *Salmonella* isolates for the same antimicrobial panel used for *Campylobacter*. Forty-seven (*n* = 47) *Salmonella* isolates from the whole carcass washing were used in the MIC assay, only changing the antimicrobial concentrations: gentamicin (0.125–128 µg/mL), ciprofloxacin (0.063–64 µg/mL), nalidixic acid (0.125–1024 µg/mL), and tetracycline (0.025–256 µg/mL). The inoculums were prepared in the same method to 0.5 McFarland Standard by suspension in sterile distilled water, and 10 µL of the prepared inoculum was added into all the wells except the growth control and incubated at 37 °C for 20 h. Plates were read against a dark background. The MIC was the lowest concentration of antimicrobials that inhibited bacterial growth. The strains were classified as susceptible or non-susceptible (including intermediate strains) according to the breakpoints described either in the CLSI standards or epidemiological MIC cut-off (ECOFF) values of EUCAST breakpoints [33,34,35]. ATCC 29,213 *Staphylococcus aureus* was used as a quality control strain.

### 2.5. Statistical Analysis

The data obtained for the microbiological counts are presented as mean ± standard error. One-way analysis of variance (ANOVA) followed by Tukey’s multiple comparison test was used to determine statistical differences in the effects of chlorine on the *Campylobacter* bacterial load. Prevalence data were analyzed using Fisher’s exact test and one sample *t*-rest. All statistical analyses were performed using either SPSS Version 25 (IBM, Armonk, NY, USA) or GraphPad Prism Version 8 (GraphPad Software, Inc., Boston, MA, USA). In all cases, a *p*-value of <0.05 was considered statistically significant.

## 3. Results

### 3.1. Enumeration of Campylobacter and Salmonella

In total, 150 whole chicken carcass washings, 100 neck skin samples, and 100 ceca samples were collected. The overall prevalence of *Salmonella* was 78.25%, while *Campylobacter* was 63.2%. There was no significant difference between the positive percentages and sample types for both *Salmonella* and *Campylobacter* (Table 3).

**Figure 1:**
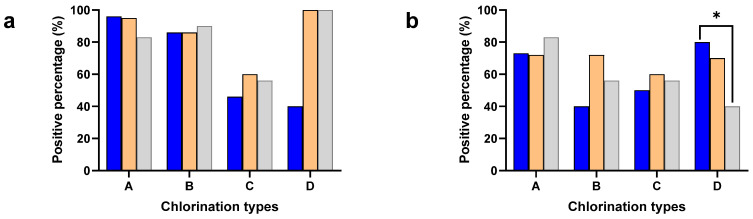
Positive percentages of neck skin (Blue), whole carcass (Orange), and ceca (Gray) for *Salmonella* (**a**) and *Campylobacter* (**b**) in plant types with different chlorine concentrations: 3–5 ppm (A), 20–30 ppm (B), 40–50 ppm (C), and sodium metabisulphite (D). * Denotes statistically significance (*p* < 0.05).

The *Salmonella* and *Campylobacter* positive percentages were separately calculated to understand the effect of different chlorine concentrations in the chill tank on minimizing cross-contamination (Figure 1). The neck skin samples were collected after washing with potable water (2–3 ppm of chlorine) except plant type D (without chlorination), while the chicken carcasses had undergone chemical sanitization in the chill tank, either with chlorine (A, B, C) or other sanitizers (D).

The carcass contamination was significantly higher compared to the neck skin in both *Salmonella* (*p* < 0.001) and *Campylobacter* (*p* < 0.05) in the chill tank irrespective of the plant type, where different chlorination techniques were used. Especially, Type D used sodium metabisulphite in the chill tank and detected significantly higher (*p*< 0.001) *Salmonella* contamination (100%), compared to the neck skin samples (40%). A significant difference was not observed between the plant types with different chlorine concentrations either in the positive percentages of the neck skin or whole carcasses.

**Figure 2:**
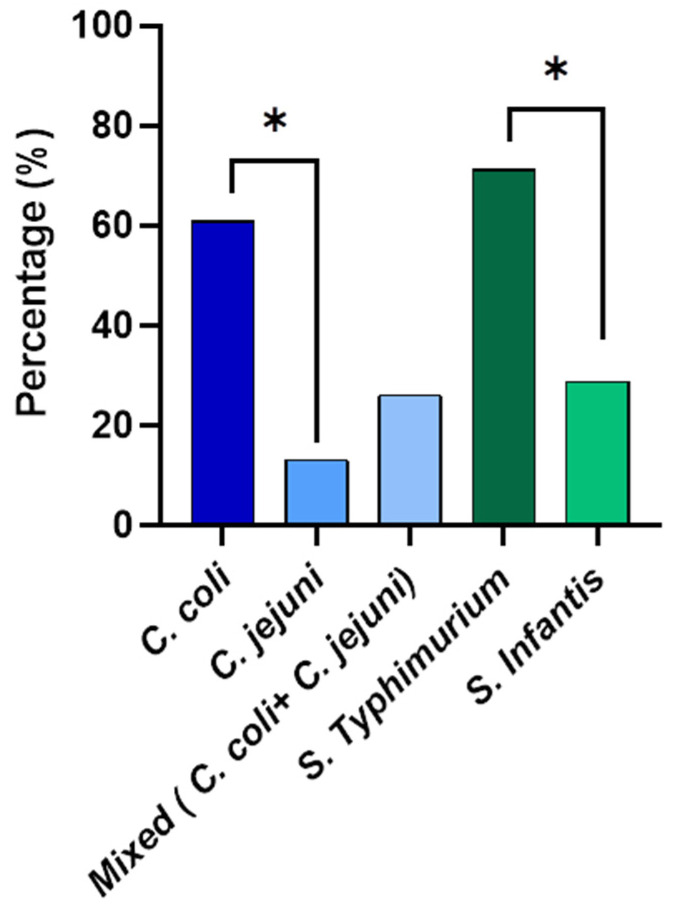
The prevalence of *Campylobacter* spp. and non-typhoidal *Salmonella* spp. enumerated from the whole chicken carcasses. * Denotes statistically significance (*p* < 0.05).

Species identification was performed for *Campylobacter* and *Salmonella* isolated from the whole carcass washings (Figure 2). The qPCR results revealed that out of the isolated *Campylobacter* from the whole carcasses, 60.9% was *Campylobacter coli* and 13% was *Campylobacter jejuni*. Interestingly, 26.1% were mixed cultures with both *C. coli* and *C. jejuni*. The prevalence percentage of *C. coli* was significantly higher than (*p* < 0.05) that of *C. jejuni*. According to the serotyping results, the prevalence of *S.* Typhimurium was 71.1% and 28.8% of the isolates were *S.* Infantis from the whole chicken carcass isolates. The *S.* Typhimurium prevalence percentage was significantly higher (*p* < 0.05) than that of *S.* Infantis.

Positive percentages of cecal samples for *Salmonella* and *Campylobacter* were included in the analysis to understand the flock prevalence of *Salmonella* and *Campylobacter*, where the birds are entering into the processing lines of the identified different processing plant types: A, B, C, and D (Figure 1). A significant difference was not observed in either the *Salmonella* or *Campylobacter* positive percentages in the present study. However, the positive percentage of *Salmonella* (79%) was higher compared to *Campylobacter* (63%) in the ceca. The significant difference between the positive percentages of the ceca and whole carcass for *Salmonella* was not observed in the present study. Interestingly, the positive percentage of the neck skin for *Campylobacter* was significantly higher (*p* < 0.05) compared to the ceca in Type D.

**Figure 3:**
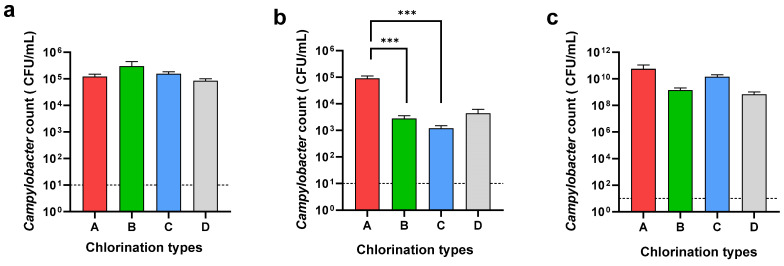
*Campylobacter* counts of neck skin (**a**), whole carcass (**b**), and ceca (**c**) in different chlorination types: 3–5 ppm (A), 20–30 ppm (B), 40–50 ppm (C), and sodium metabisulphite (D). *** Denotes statistically significance (*p* < 0.001).

The *Campylobacter* count of the neck skin and whole carcass washing was determined to understand the effect of chemical decontamination in the chill tank. Further, the *Campylobacter* counts in the cecal samples were determined to understand the *Campylobacter* load entering the processing line in each plant type (Figure 3).

While the *Campylobacter* loads on the neck skin were almost similar in all four types, A, B, C, and D, the *Campylobacter* loads on the whole carcasses were different. A significant difference (*p* < 0.001) was observed in the *Campylobacter* load on the carcasses when using 20–30 ppm (2.8 × 10^3^ CFU/mL) and 40–50 ppm (1.3 × 10^3^ CFU/mL) of chlorine compared to 3–5 ppm (9 × 10^4^ CFU/mL) of chlorine in the chill tank. There was no significant difference between the counts on the whole carcass when using either 20–30 ppm or 40–50 ppm of chlorine in the chill tank. Although the usage of sodium metabisulphite reduced the level of *Campylobacter* (4.3 × 10^3^ CFU/mL) on the whole carcasses, the reduction was not significant compared to 3–5 ppm chlorine. When comparing the *Campylobacter* reduction in the whole carcasses in the chill tank compared to the neck skin, an average 2-log reduction was observed, at a 20–30 ppm and 40–50 ppm chlorine concentration in the chill tank. In Type D (sodium metabisulphite), only a 1-log reduction was observed in the whole carcasses compared to the neck skin. As an average, the cecal *Campylobacter* load entered into the processing plants in the present study was as high as 1.83 × 10^10^ CFU/mL. The average cecal *Campylobacter* counts in plant types A, B, C, and D was 5.65 × 10^10^ CFU/mL, 1.4 × 10^9^ CFU/mL, 1.4 × 10^10^ CFU/mL, and 7 × 10^8^ CFU/mL, respectively. There was no significant difference in the *Campylobacter* load entering into the processing lines of the selected processing plants.

### 3.2. Minimum Inhibitory Concentration (MIC) for Campylobacter and Salmonella

A micro broth dilution test was performed to determine the MIC levels of antimicrobials to understand the antimicrobial resistance of *Campylobacter* and *Salmonella* isolated from whole chicken carcasses. Four types of antimicrobials, gentamicin, ciprofloxacin, nalidixic acid, and tetracycline, from different antimicrobial classes, which are commonly used in the poultry industry and targeted at *Campylobacter* and *Salmonella* AMR surveillance programs, were used in the MIC test. The MIC results and antimicrobial resistance profiles are described in Table 4 and Table 5.

**Figure 4:**
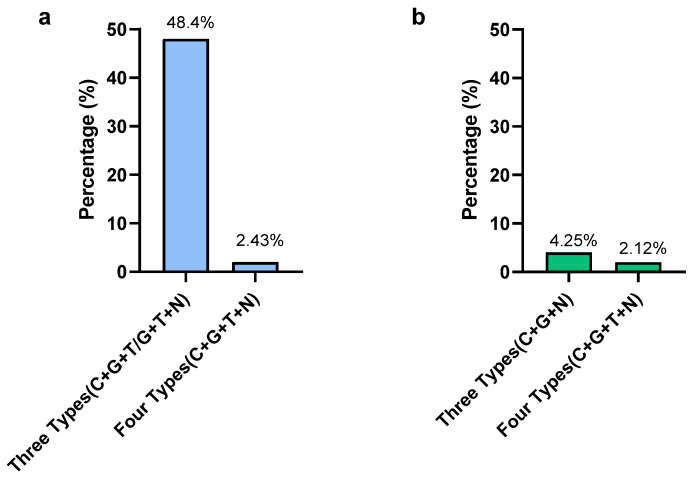
Prevalence of multidrug-resistant *Campylobacter* (**a**) and *Salmonella* (**b**) in whole chicken carcasses. Gentamicin (G), ciprofloxacin (C), nalidixic acid (N), and tetracycline (T).

In *Campylobacter*, according to the MIC results, the highest resistant percentages were observed for gentamicin and ciprofloxacin, which were 87.8% and 68.3%, respectively. The lowest resistance was observed in nalidixic acid (7.31%). Only two isolates were susceptible to all types of antimicrobials used in this study and those two were *C. jejuni*. Out of 41 isolates, 20 isolates (48.7%) were detected as resistant to 3 types of antimicrobials (C + G + T/G + T+N) (Figure 4). Only one *C. coli* isolate (2.42%) was resistant to all four types of antimicrobials. From a total of 41 *Campylobacter* isolates, 21 isolates (51.2%) showed multidrug resistance by being resistant to more than 3 classes of antimicrobials. The present study revealed that 75% of MDR *Campylobacter* spp. in the whole carcass washings were *C. coli.*

In *Salmonella*, the highest resistance was observed for tetracycline (63.8%) and nalidixic acid (36.2%). The lowest resistance was observed in gentamycin (8.5%). Out of 47 isolates, only 1 (2.12%) *S.* Typhimuriumisolate was resistant to all 4 types of antimicrobials, while 2 *S.* Typhimurium isolates (4.25%) were resistant to 3 types of antimicrobials (gentamicin, ciprofloxacin, nalidixic acid) (Figure 4). The MDR of *Salmonella* for the selected antimicrobials in the present study was 2.12%. The MDR of *Salmonella* spp. detected in the present study was *S.* Typhimurium, while all *S.* Infantis isolates were sensitive to selected antimicrobials except only one, which was resistant to nalidixic acid.

In the present study, *Campylobacter* showed a higher resistance to gentamicin and ciprofloxacin, while *Salmonella* showed a higher resistance to nalidixic acid and tetracycline.

## 4. Discussion

Consumption of chicken meat contaminated with foodborne pathogens such as *Salmonella* and *Campylobacter* remains a significant public health risk worldwide. Despite many interventions implemented in processing plants to minimize the bacterial load, the persistence of foodborne pathogens in chicken meat is a continuous burden in the industry. Although the European Union banned using chlorine in poultry processing [36], using chlorinated water in both inside–outside carcass washers and chill tanks is a common practice in Sri Lanka and many other countries in the world [27,37]. The variations in chlorine concentrations used in the chill tanks create a significant risk of persisting foodborne pathogens in the production chain. In the present study, mainly three types of poultry processing plants were identified according to the different chlorine concentrations used in the chill tank, which ranged from 2 ppm to 50 ppm. Apart from that, one processing plant used sodium metabisulphite instead of chlorine, which is usually used in water treatment plants [38]. As this is the first comprehensive study conducted in Sri Lanka to determine the *Salmonella* and *Campylobacter* contamination in the poultry processing line, we tried to understand the differences in the bacterial contamination levels in different processing plant types.

The overall prevalence of *Salmonella* was 78.5%, while that of *Campylobacter* was 63.7%. The present prevalence levels of both pathogens were significantly higher than the previous studies conducted in Sri Lanka [18]. Due to the financial crisis following COVID-19 in Sri Lanka, the increased production cost has badly affected the poultry industry. Therefore, the financial affordability for infrastructure and biosecurity facilities has been limited and infection rates might be increased. Furthermore, the flock ages have increased; hence, the colonization and spreading might be increased. The intestinal colonization of *Salmonella* and *Campylobacter* may increase with the age of the flock [39]. The present results stand with a previous study conducted to understand the weather correlation in *Campylobacter* prevalence in Sri Lanka [40]. Notably, the *Salmonella* contamination percentages in the ceca, neck skin, and whole carcass were significantly higher (*p* < 0.05) compared to the *Campylobacter* contamination percentages of the same samples. This could be due to the differences in colonization, shedding, stress response mechanisms, and survivability under the chlorine treatment in *Salmonella* [41,42]. Previous studies have shown that the persistence of *Salmonella* in chicken meat is higher compared to *Campylobacter* [43]. Unlike *Salmonella, Campylobacter* is a very fastidious organism that cannot easily survive in harsh environments [44]. Another major concern is that the ability of biofilm formation of *Salmonella* on processing equipment is high. Moreover, *Salmonella* in biofilms are more resistant to antimicrobials and they can survive during cleaning and disinfection and persist for a long period, which may increase product contamination [45]. To understand the cecal colonization of foodborne pathogens, the positivity was determined. The prevalence of *Campylobacter* in the ceca was 63%, which is very similar (67.6%) to a previous study conducted in broiler flocks [16]. However, the overall prevalence of *Salmonella* was lower than the previous study conducted in Malaysia [46], while the *Salmonella* prevalence in the cecal samples was significantly higher in the present study compared to the study conducted in India [47]. This could be due to differences in infection pressures and the isolation techniques.

All the processing plants selected in the present study used potable water (2–3 ppm) in the inside–outside carcass washers, except type D, which used water without chlorination. The results revealed that, even after the carcass washing, the *Campylobacter* and *Salmonella* contamination rates of the neck skin (57% and 73%) samples were higher than the previous studies, where a 27.4% *Campylobacter* and 21.4% *Salmonella* contamination in semi-automated poultry processing plants were reported [16,48]. The increased carcass contamination in the present study could be due to the visceral rupture during evisceration, which inevitably leads to the contamination of equipment, working surfaces, and water [49]. The high contamination rate of the neck skin in the present study highlighted the incorrect evisceration techniques and the inefficiency of using carcass washers to reduce the contamination levels [50]. Although the inside–outside carcass washers remove the fecal materials and tissue debris, they have limited effectiveness in reducing the bacterial load in poultry carcasses [51]. The bacterial load on the carcasses was expected to be reduced in the chill tank by washing, chemical decontamination, and chilling. However, the whole carcass contamination of *Salmonella* and *Campylobacter* in the chill tank in the present study was 80.66% and 68.66%, respectively. It was significantly higher than the previous study conducted in Sri Lanka, which was reported as 10% and 32%, respectively [18]. Our finding stands with a study conducted in retail shops, where a 59% prevalence of *Campylobacter* was reported [16]. The detected high *Campylobacter* cecal colonization (1.83 ×10^10^ CFU/mL) in the present study ensured the entry of high loads of *Campylobacter* into the processing line, which can increase the risk of carcass contamination [46,47]. A positive correlation has been observed between the contamination of carcasses and the high positivity rates for *Campylobacter* of flocks at the farm level [52]. Therefore, farm intervention by increasing biosecurity to reduce colonization is also very important to reduce carcass contamination [53]. Moreover, due to the rapid horizontal transmission of *Campylobacter,* the flock prevalence can reach up to 100% within a few days [44]. Notably, the lack of clinical signs causes difficulty in measures to reduce flock prevalence [54]. Furthermore, previous studies demonstrate that even though the flock prevalence of *Salmonella* is low, the cross-contamination during processing leaves the plant with significantly higher carcass contamination [55]. The contaminated carcasses enter the chill tank, facilitating the cross-contamination of non-infected carcasses [56]. Notably, in the present study, as there was no significant difference observed in the cecal *Campylobacter* counts between each plant type, the different contamination levels of whole carcasses could be due to the differences in carcass contamination and the effect of the chlorine concentrations. The positive percentages of *Salmonella* (80.66%) and *Campylobacter* (66.66%) in the whole carcasses were significantly higher (*p* < 0.001 and *p* < 0.05) compared to the neck skin irrespective of the chlorine concentrations. This could be due to the cross-contamination in the chill tank. Notably, the prevalence of *C. coli* (60.9%) in the whole carcasses was significantly (*p* < 0.05) higher than *C. jejuni* (13%), while 26.1% was detected as mixed cultures with both *C. coli* and *C. jejuni.* This study was the first evidence of reporting *Campylobacter* species identification in whole chicken carcasses in Sri Lanka. The other countries have reported the most common *Campylobacter* spp. is either *C. jejuni* [57] or *C. coli* [58], which were considered as the most common *Campylobcater* spp. cause in over 95% of human infections [44]. The prevalence of *S.* Typhimurium in the present study (71.1) was higher than the previously reported 47.8% in chicken meat in Sri Lanka [26]. *S.* Infantis (28.8%), which also causes human infections, was revealed in the present study and was not previously reported in chicken meat in Sri Lanka. *S.* Infantis is becoming an increasingly prevalent serovar globally [59,60]. Worldwide, *S.* Infantis is reported as the most common serovar isolated from animal and food sources, with the majority of strains originating from broilers [61].

The positive percentages of the whole carcasses could be increased significantly (*p* < 0.05) after evisceration [62]. Even after using either 20–30 ppm or 40–50 ppm of chlorine in the chill tank, the *Salmonella*-contaminated carcasses’ percentage was not reduced. This could be due to increased organic matter contents (such as residual fecal material, blood, skin, or feathers), which reduce the availability of free chlorine in the solution [63]. Similarly, a high *Salmonella* load entering the chill tank could be another reason for the reduced effectiveness of chlorine [57]. A previous study has shown that there is no significant difference in using either water or chlorine in the chill tank to reduce either *Campylobacter* or *Salmonella* [64]. Interestingly, *Salmonella* positivity in the whole carcasses (100%) was significantly higher (*p* < 0.05) compared to the neck skin samples (40%), where sodium metabisulphite was used in the chill tank. Noteworthy, the incidence of contamination increases from pre-chill to post-chill due to the cross-contamination during processing [55]. Moreover, the obtained results could be due to the lower effectiveness of sodium metabisulphite in reducing the bacterial levels. Importantly, the effectiveness of a disinfectant always depends on the type of active ingredient, concentration, and time of exposure [64]. Anyway, further studies have to be conducted to understand the effect of sodium metabisulphite in reducing the bacterial load in the carcass. Similarly, the carcass contamination with *Campylobacter* was also high (70%), either for the 20–30 ppm (Type B) or 40–50 ppm (Type C) chlorine concentrations. Although the European Union has banned the use of chlorine in food processing, the WHO has recommended the use of 50–70 ppm with 0.4–4 ppm free available chlorine (FAC) in chiller water. A recent in vivo study has demonstrated that *Campylobacter* needed at least 128 ppm of chlorine in complete inactivation with irreversible cell damage [57]. Although chlorine is considered a fast oxidative agent that damages both the cell membrane and the cytoplasm [65], lower efficacy has been reported compared to other sanitizers [54]. This could also be due to the enhanced survivability of *Campylobacter*, by inducing the adaptive stress response mechanism under the chemical stress caused by chlorination [66].

In the present study, we tried to understand the reduction in the *Campylobacter* load in the whole carcasses by chemical decontamination in carcass washing. The *Campylobacter* load in the neck skin was almost similar in all four types of processing plants: A, B, C, and D with an average of 1.4 × 10^5^ CFU/mL, which could be due to the same intervention of carcass washing of using potable water for inside–outside carcass washers. According to the results obtained, a similar cecal *Campylobacter* load is entering into the processing lines of all four types of processing plants. Moreover, the *Campylobacter* counts in the neck skin samples are also more or less similar. Therefore, the carcass contamination during evisceration can be predicted as more or less similar. Hence, the limitation caused by the effect of the evisceration technique could be ignored in the present study. Therefore, the differences in the *Campylobacter* counts on the whole carcasses could be due to the variations in the effectiveness of the chlorine concentrations. As expected, the *Campylobacter* load was significantly low (*p* < 0.001) in the whole carcass, where 20–30 ppm (2.8 × 10^3^ CFU/mL) and 40–50 ppm (1.3 × 10^3^ CFU/mL), compared to the 3–5 ppm chlorine in the chill tank. *Campylobacter* needs higher chlorine concentrations in their inactivation [57]. Further, an average 2-log reduction in the *Campylobacter* count was observed in the whole carcass compared to the neck skin after the chemical decontamination of the carcasses in the chill tank. A risk assessment study has shown that by reducing the *Campylobacter* load on raw poultry by 2-log units, human campylobacteriosis can be reduced by 30-folds [66]. A reduction in the *Campylobacter* load in chicken meat is very important as the infection dose of *Campylobacter* is very low and ingestion of 500–1000 cells can cause human infection [17]. However, the sub-lethal injury and formation of viable but non-culturable (VBNC) form of *Campylobacter* after exposure to a sanitizer reduces the detectable count, while the persistence in the food chain is significantly high [57,67]. Importantly, the expression of virulence genes in sub-lethally injured *Salmonella* and *Campylobacter* after exposure to chlorine has been demonstrated in a previous study [43]. Therefore, entering these sub-lethally injured foodborne pathogens after exposure to chlorine into the food chain is a serious public health risk.

With the high prevalence of *Campylobacter* and *Salmonella* on the whole chicken carcasses in the present study, it is important to understand their resistance patterns to commonly used antimicrobials in the poultry industry. *Campylobacter* and *Salmonella* are colonized in the chicken gut together and can cause co-infection in birds; more or less similar resistant patterns can be developed in both pathogens for the antimicrobials used in infection control, which could be a serious public health issue. The results of the present study revealed that gentamicin has the highest level of resistance (87.8%) in *Campylobacter*, which was higher than in the previous studies reported for gentamicin of 10% [16]. The ciprofloxacin resistance was 68.3% and it was lower than the previously reported 80% in the same study. *Campylobacter* is increasingly resistant to antibiotics, especially fluoroquinolones and macrolides, which are the most frequently used antimicrobials for the treatment of campylobacteriosis when clinical therapy is required. Fluoroquinolones are considered the second-line treatment against human campylobacteriosis [22]. Therefore, the emergence of fluoroquinolone-resistant *Campylobacter* could be a major risk in human antimicrobial treatments in the future. The observed resistance to ciprofloxacin could be due to the tremendous use of enrofloxacin in the poultry industry in Sri Lanka, which is structurally related to ciprofloxacin and shares the same resistant mechanism [68]. Interestingly, the resistance to nalidixic acid in the present study was very low (7.31%) compared to the previous study, which reported the resistance as 80% [16]. A study conducted in Brazil revealed that a 90.7% ciprofloxacin and 81.5% nalidixic acid resistance in *Campylobacter* [29]. In contrast, the tetracycline resistance percentage of *Campylobacter* in the present study was lower than the previously reported studies [16]. This could be due to the strain variation, differences in the sample numbers, and differences in antimicrobial usage. Therefore, the patterns and practices of antimicrobial usage in food animals can determine the development of antimicrobial resistance in foodborne pathogens such as *Campylobacter* and *Salmonella*. As human campylobacteriosis is a highly travel-associated infection, travelers to Asia have been shown to carry resistant *Campylobacter*, which reflects the above situation [69]. Interestingly, only two isolates of *C. jejuni* were susceptible to all antimicrobial classes used in the present study, which stands with the results of a previous study conducted with the same antimicrobials [69]. Importantly, from the *Campylobacter* isolates, 48.7% were resistant to three types of antimicrobials either for a tetracycline, ciprofloxacin, and gentamycin combination or a tetracycline, gentamycin, and nalidixic acid combination. The observed higher multidrug resistance (51.2%) in *Campylobacter*, isolated from the whole carcass washing, in the present study showed the persistence of multidrug-resistant *Campylobacter* in the chilled chicken of the processing plants. The observed MDR was higher than the previous study, which was reported as 13% [19]. Notably, it was revealed that 75% of MDR *Campylobacters* are *C. coli*. This could be due to the higher prevalence of *C. coli* in the present study. Therefore, the persistence of MDR *Campylobacter coli* in poultry processing lines in Sri Lanka might be an alarming situation for future antimicrobial usage in both livestock and human medicine.

Similarly, *Salmonella* has shown a higher resistance to tetracycline (63.8%) and nalidixic acid (36.2%) in the present study. This could be due to the greater usage of tetracycline and quinolone in the poultry industry in Sri Lanka. This result stands with the results observed in a previous study conducted for poultry *Salmonella* [70]. Also, the resistant levels and the patterns are in agreement with a previous study conducted in Iran, which revealed that the majority of the *Salmonella* isolates were resistant to nalidixic acid, tetracycline, and streptomycin [71]. The resistance patterns associated with *Salmonella* to the important therapeutic antimicrobials used in human medicine, such as tetracycline and fluoroquinolones, have to be considered seriously. All the MDR *Salmonella* spp. reported in the present study were *S.* Typhimurium. This could be due to *S.* Typhimurium being the most common *Salmonella* spp. recovered from the carcass washings. The resistant traits of *Salmonella* serotypes in Sri Lanka were reported in a previous study [72]. The increased prevalence of multidrug-resistant *S.* Typhimurium in South Asian countries was reported previously [73]. Interestingly, all *S.* Infantis isolates, except only one, from the whole carcass washings were sensitive to the selected antimicrobial in the present study, which was resistant to nalidixic acid. CDC is more concerned about *S.* Infantis, as an emerging human multidrug-resistant pathogen [74]. Antimicrobials are used more extensively in *Salmonella* than *Campylobacter* as it causes clinical infection in poultry. As the prevalence of *Salmonella* in the chicken carcass was significantly high in the present study, the risk of transmission of resistant genes into the human population is significant. Nevertheless, the higher antimicrobial resistance observed in *Campylobacter* than in *Salmonella* could be due to the strain variation and induced antimicrobial resistance in *Campylobacter* after exposure to chlorine [43,75]. The present study emphasizes that the importance of *Campylobacter* as the worldwide number one cause of gastroenteritis infections in humans could be a more serious public health issue in the future [76].

However, previously, using a high heat, long duration, and more spices in cooking might destroy most of the contaminated bacteria in meat [46]. The lifestyle, cooking patterns, and eating habits of Sri Lankans have changed drastically during the last few decades. Therefore, the risk of consuming partially cooked chicken meat has increased in the present than in the past in Sri Lanka. While minimizing cross-contamination in the poultry processing plants via chemical decontamination, reducing the bacterial load entering into the processing line via infected birds also has a similar importance in preventing cross-contamination. Implementation of strict biosecurity protocols, with proper vaccination, proper nutrition, and disease surveillance programs, helps to reduce infection pressure in poultry farms [77]. Moreover, historically, chlorine has been the industry standard for decontaminating chicken meat during processing other food-grade sanitizers such as acidified sodium chlorite and peracetic acid, which have replaced chlorine as primary antimicrobials in other developed countries [68].

## 5. Conclusions

To the best of our knowledge, this is the first report on the prevalence of *Campylobacter coli, Campylobacter jejuni,* and *Salmonella* Infantis in processed chicken carcasses in Sri Lanka and their antimicrobial resistance patterns. The present study showed that although the high chlorine concentrations have reduced the bacterial load, the persistence of non-typhoidal *Salmonella* spp. and *Campylobacter* spp. in poultry processing lines is significantly high. Therefore, it is important to implement an effective chemical decontamination strategy to minimize cross-contamination along with a proper disease control program in poultry farms. Furthermore, the emergence of multidrug-resistant *Campylobacter* and *Salmonella* is a major public health risk. Moreover, resistance to fluoroquinolones could be a future risk in treatment options in humans. Implementing a national antimicrobial stewardship program to minimize the misuse of antimicrobials in the poultry industry might help mitigate AMR in foodborne pathogens in Sri Lanka. In addition, extensive molecular study is urgently needed to identify epidemic clones that carry multiple resistant genes such as MDR. The whole genomic approach may fill existing gaps shortly.

## Figures and Tables

**Table 1:** DNA sequences of the primers used for the detection of *Campylobacter* spp.

Primer Name	Sequences (5′–3′)	Target Gene
*C.jejuni*-F	ATGAAGCTGTGGATTTTGCTAGTG	*hipO*
*C.jejuni*-R	AAATCCAAAATCCTCACTTGCCA	*hipO*
*C.coli*-F	CATATTGTAAAACCAAAGCTTATC	*glyA*
*C.coli*-R	AGTCCAGCAATGTGTGCAATG	*glyA*

**Table 2:** The specified antigen formulae of *Salmonella* serovars in Kauffman–White reference catalogue.

Group	Serova	Somatic (O) Antigen	Flagella (H) Antigen
O: B	*Salmonella enterica subspecies enterica serovar* Typhimurium	1,4,5,12	i (phase 1)
O: C1	*Salmonella* enterica subspecies enterica serovar Infantis	6,7,14	r (phase 1)
O: D	*S. enterica* subspecies *enterica serovar* Enteritidis	1,9,12	g,m (phase 1)

**Table 3:** Prevalence of *Salmonella* and *Campylobacter* in poultry processing plants.

Sample Type	Sample Number	*Salmonella* Positive Percentage (%)	*Campylobacter * Positive Percentage (%)
Carcass wash	150	80.66 (121/150)	68.66 (103/150)
Neck skin	100	73 (73/100)	57 (57/100)
Ceca	100	79 (79/100)	63 (63/100)
Total	350	78.25 (273/350)	63.7 (223/350)

**Table 4:** Minimum inhibitory concentration and antimicrobial susceptibility of *Campylobacter*.

Antimicrobial Agent					Minimum Inhibitory Concentration (MIC) *n* (%)											Resistant %
	0.076	0.0312	0.063	0.125	0.25	0.5	1	2	4	8	16	32	64	124	256	
GEN *		0	0	0	0	1	3	1	1	1	4	30				
						(2.4)	(7.3)	(2.4)	(2.4)	(2.4)	(9.8)	(73.2)				87.8
CIP	2	1	1	1	1	3	1	3	6	8	14					
	(4.9)	(2.4)	(2.40	(2.4)	(2.4)	(7.3)	(2.4)	(7.3)	(14.6)	(19.5)	(34.1)					68.3
NAL				0	0	0	1	1	2	3	4	27	2	1	0	
						(2.4)	(2.4)	(2.4)	(4.9)	(7.3)	(9.8)	(65.9)	(4.9)	(2.4)		7.31
TET	0	0	0	0	3	1	0	2	4	15	16					
					(7.3)	(2.4)	0	(4.9)	(9.8)	(36.6)	(39)					39

Gentamicin (GEN), ciprofloxacin (CIP), nalidixic acid (NAL), and tetracycline (TET). Reference values are based on *Campylobacter jejuni*/*coli* breakpoints from CLSIM45, 3rd Ed. * Reference values are based on *Campylobacter jejuni/coli* epidemiological MIC cut-off (ECOFF) values (EUCAST breakpoints (https://www.eucast.org/fileadmin/src/media/PDFs/EUCAST_files/Breakpoint_tables/v_14.0_Breakpoint_Tables.pdf, accessed on 1 June 2024). Continuous lines indicate CLSI/CAST breakpoints. Shaded areas indicate the tested concentrations of antimicrobials.

**Table 5:** Minimum inhibitory concentration and antimicrobial susceptibility of *Salmonella*.

Antimicrobial Agent					Minimum Inhibitory Concentration (MIC) *n* (%)											Resistant %
	0.063	0.125	0.25	0.5	1	2	4	8	16	32	64	128	256	512	1028	
GEN *		0	4	7	14	6	9	3	3			2				
			(8.5)	(14.8)	(29.7)	(12.7)	(19.1)	(2.4)	(6.3)			(4.2)				8.5
CIP		27	6	7	2	3	0	0	0	2	0					
		(57.4)	(12.7	(14.8)	(4.2)	(2.4)				(4.2)						14.9
NAL		0	0	0	0	6	4	14	6	7	7	3				
						(12.7)	(8.5)	(29.7)	(12.7)	(14.8)	(14.8)	(6.3)				36.2
TET			0	2	2	1	6	6	16	13	0	0	1			
					(4.2)	(2.1)	(12.7)	(12.7)	(34)	(27.6)			(2.1)			63.8

Gentamicin (GEN), ciprofloxacin (CIP), nalidixic acid (NAL), and tetracycline (TET). * Reference values are based on Enterobacterales breakpoints from CLSIM100, 33rd Ed. Continuous lines indicate CLSI/CAST breakpoints. Shaded areas indicate the tested concentrations of antimicrobials.

## Data Availability

All the data are available with the corresponding author.
